# Pharmacologic Considerations for Antimicrobials and Anticoagulants after Burn Injury

**DOI:** 10.3390/ebj4040038

**Published:** 2023-11-10

**Authors:** Pranav Ravichandran, Kaitlin A. Pruskowski

**Affiliations:** 1 F. Edward Hebert School of Medicine, Uniformed Services University of the Health Sciences, Bethesda, MD 20814, USA; 2 United States Army Institute of Surgical Research, JBSA Fort Sam Houston, San Antonio, TX 78234, USA

**Keywords:** burn, critical illness, pharmacokinetics, pharmacodynamics, coagulopathy, post-burn management, post-burn antibiotic dosing

## Abstract

Derangements in pharmacokinetics and pharmacodynamics (PK/PD) of burn patients are poorly understood and lacking consistent data. This leads to an absence of consensus regarding pharmacologic management of burn patients, complicating their care. In order to effectively manage burn critical illness, knowledge of pharmacologic parameters and their changes is necessary. It is also imperative that the clinician understands how these changes will affect drug dosing. A common practice is to increase antibiotic dosing and/or frequency; however, this may not be necessary and doses should be adjusted to patient- and drug-specific parameters. Additionally, monitoring assays for antibiotic levels as well as coagulation factors can be useful for adjusting dosages to best treat the patient. This review focuses on alterations in PK/PD as well as other physiologic changes after burn injury, with special reference to care in military and austere settings.

## 1. Introduction

Burn patients present with a unique alteration to the body’s physiologic mechanisms on top of the already complex level of care required of any trauma patient. These patients require multidisciplinary care to manage the varying needs of the burn patient who shows an increasingly dynamic recovery. As recovery progresses, the physiologic principles informing management can shift dramatically. This requires providers to adjust management plans quickly and may lead to increased difficulty in treating common ICU issues such as pneumonia and shock. To successfully manage post-burn critical illness as well as its complications, it is crucial to understand the recovery dynamics in burn patients in order to help tailor pharmacologic treatments in response to their ongoing physiological changes.

Infection is a common complication of any ICU patient. Burn patients are particularly susceptible to these infections due to dysregulation of the immune system. Post-injury infection, such as pneumonia, urinary tract infections, and sepsis, is the leading cause of death for burn patients [1]. Additionally, local and systemic inflammation creates metabolic and vital sign derangements typically seen with infection, such as tachycardia, tachypnea, or fever. Therefore, the inflammatory response in burn patients obscures the classic signs of infection or sepsis, making it difficult for providers to diagnose. As such, most burn patients already meet at least one systemic inflammatory response syndrome (SIRS) criteria, rendering it ineffective for diagnosing an infection. The deployed environment poses significant challenges for infection prevention and management. Casualties who sustain burns during military operations have been shown to be more likely to die from infectious complications, including fungal and Gram-negative organisms [2]. Multidrug resistant organisms (MDRO) have been shown to have a high prevalence in military burn casualties [3]. Limited resources, including systemic and topical antimicrobials, and delayed definitive surgical management may contribute to infection. 

Coagulopathy from burn injuries is another common complication in the care of burn patients. Burn trauma often activates the coagulation cascade in the early post-burn phase, leading to a pro-coagulable state. This state is likely due to cytokine-mediated cascade activation via the hyperinflammatory response and tissue factor activation [4]. Because of this activation, these patients often meet all three criteria of Virchow’s triad (immobilization, endothelial injury, and hypercoagulability), predisposing these patients to venous thromboembolism formation (VTE). This propensity for coagulation with the previously mentioned physiologic alteration then complicates VTE management, with many patients not having adequate prophylaxis [5,6]. It is worth noting that burn patients can become either hypercoagulable from excess cascade activation or hypo-coagulable due to dysfunctional clotting factors and platelets. In one military burn center, the incidence of VTE was greater than 2%, a finding that was similar to that of combat casualties [7,8]. Prolonged transport from point of injury to definitive care, along with the hypercoagulable state seen after burn injury, may contribute to VTE risk in patients burned during military operations.

The goal of this article is to present healthcare providers with a review of background and current literature to guide pharmacologic management decisions in burn patients. Recognizing and understanding how alterations in physiology in burn patients affect pharmacologic dosing is important for the best possible outcomes in recovery.

## 2. Pharmacokinetics (PK) and Pharmacodynamics (PD) 

### 2.1. Overview

As previously mentioned, it is crucial to understand pharmacokinetic and pharmacodynamic parameters to optimize management of patients with critical illness such as burn injury. Pharmacokinetics refers to the parameters of absorption, distribution, metabolism, and elimination of administered medications. Pharmacodynamics represents the response to a drug once it is has reached the target or its site of action. Use of pharmacologic agents depends on these parameters and changes frequently in critical illness patients due to the patient’s altered hepatic, renal, and GI physiology. This review will focus on these principles and how they change in response to critical illness in order to guide management decisions.

Absorption is the process by which drug molecules move from the administration site to the vasculature and then to the organs and tissues. An important variable of medication is bioavailability (F), defined as the fraction of medication in systemic circulation. Absorption is primarily related to the route of administration, i.e., intramuscular, subcutaneous, enteral, topical, etc. Clinically, absorption is affected by characteristics at the site of administration, such as pH, blood flow, first-pass metabolism, and motility.

Distribution, or volume of distribution (V_d_), describes the relationship between dosage of a drug and the serum concentration. Drug characteristics influence the volume of distribution and its ability to penetrate tissue; a more lipophilic molecule will have a higher V_d_ whereas a hydrophilic molecule will have a lower V_d_. It is important to remember that serum protein levels, fluid status, and large-volume resuscitation after burn injury will also affect distribution.

Metabolism refers to the conversion of drug molecules to either active or inactive metabolites. While this occurs in many bodily tissues, the primary organ of concern is the liver. Decreased hepatic blood flow or injury to the liver parenchyma can directly affect the rate of metabolism, leading to the possibility of supra-therapeutic dosing or adverse drug reactions.

Elimination describes the process in which the body removes drugs from the body. Again, this occurs through various mechanisms but is primarily driven by the kidneys. K_e_ is the elimination constant that describes the rate of elimination, expressed as the fraction of drug cleared per unit time. Additionally, clearance (CL) is the volume of serum or blood cleared of a drug per unit of time and can also be used to describe elimination. Elimination is therefore dependent on renal function. In the event of poor renal function, alternative filtration methods such as hemodialysis or continuous renal replacement therapy (CRRT) often require dosage changes and can also affect clearance [9,10].

### 2.2. Alterations in PK and PD of Burn Patients

Burn patients have many physiologic changes that complicate their management. Alterations in this physiology prevent providers from adequately dosing to treat their patients. What may be guideline-directed care in other ICUs may not be enough to best treat these patients. Therefore, understanding how PK and PD change in these critically ill patients is essential. Trauma patients commonly follow an ebb and flow pattern to recovery, and this is no different in burn patients. For the first 48 h, the post-burn phase follows this ebb, characterized by burn shock and increased fluid requirements. Soon after burn injury, the resuscitative phase begins and lasts for several days. Five days after injury, burn patients enter a ‘flow’ phase, and hypermetabolism begins. This typically begins and is characterized by a surge in catecholamines, cortisol, and other hormones [11]. Adapting management requires understanding how the patient moves between these ebb and flow states and how that affects pharmacologic management.

In burn patients, especially in the early resuscitative or ‘ebb’ phase, burn shock shunts blood away from the gut and skin, thereby decreasing absorption from these systems. Enteral or subcutaneous medications can therefore be ineffective, as they may not be fully absorbed or may have delayed absorption. Any medication that has been administered can then be stored in that area and cause adverse reactions once blood flow is re-established [9]. Intravenous administration of medication bypasses this risk and increases absorption. Additionally, inflammatory processes lead to vasodilatation and increased capillary permeability, potentially moving more medication, especially hydrophilic medications, out of the vasculature.

After the medication is in the bloodstream, the body will then distribute the medication to the site of action through various mechanisms. V_d_ is primarily affected by fluid resuscitation and protein binding. In the early recovery phase, Starling forces, primarily capillary hydrostatic and interstitial oncotic pressures, adjust due to vascular hyperpermeability and decreased albumin synthesis. Burn patients are given large volumes of fluid, and along with this, relative hypoalbuminemia occurs, causing edema formation in burned and non-burned tissues. Medications that are highly bound to albumin will have an increased free fraction; this could potentially lead to adverse drug effects, especially for medications with narrow therapeutic windows (i.e., phenytoin). Additionally, edema formation may delay distribution into the target site, especially for hydrophilic medications. For these medications, larger doses or a loading dose may be needed to combat these derangements and achieve therapeutic levels. It is also important to note that the free fraction of drugs that bind to albumin may be increased due to this hypoalbuminemia, leading to toxic effects.

As the patient enters the ‘flow’ phase post-burn injury, hypermetabolism begins. The hypermetabolic phase can last up to 1–3 years after burn injury. During this phase, the patient experiences increased cardiac output, leading to faster drug distribution. Blood flow to the gut, liver, and kidneys also increases, leading to increased absorption, metabolism, and elimination. Additionally, the patient may have increased drug clearance from exudate leakage from their burn wounds. Because of these changes, the patient may need increased doses and/or more frequent dosing to achieve therapeutic serum levels. Any dose adjustments made during this time should be made based on drug- and patient-specific parameters.

## 3. The Inflammatory Response

### 3.1. Overview

Infection is a concern for any hospitalized patient and an increased level of concern is warranted for any critically ill patient. Massive trauma, large open wounds, multiple lines of access, and exposure to varying pathogens all predispose patients to inflammation and eventually sepsis. However, it is also important to note that major insults such as trauma, surgery, or burns present with similar inflammatory responses, akin to sepsis. To aid recognition of systemic inflammation, the SIRS criteria are often employed [12]. SIRS is primarily driven by signals from the innate immune system and leads to activation of various systemic responses. This occurs when local inflammation overwhelms regulatory processes [13]. Local tissue damage releases mediators such as bradykinin, serotonin, and histamines to increase vascular permeability, leading to edema. In the acute phase, IL-1, TNF-alpha, and notably IL-6 drive the manifestations of fever, leukocytosis, and increased acute-phase reactants. Complement system activation further progresses this inflammation and enacts a positive feedback loop. After reaching a threshold, the inflammation then leads to thrombus formation [13].

Simultaneous to systemic inflammation, there is the compensatory anti-inflammatory response (CARS) in the attempt to return to homeostasis and start healing [13]. This syndrome is characterized by lymphocyte apoptosis and dysfunction, hypothermia, and notably a susceptibility to infection. Like SIRS, CARS is dependent on many cytokines. IL-10 is the most important cytokine in CARS as it has many immunosuppressive roles in the body, such as TNF-alpha downregulation [14]. This is the body’s mechanism to mitigate complications from SIRS, but it does have its own major effect on the body. Immunosuppression has been suspected for many years to be a reason for patient mortality in the ICU setting. The initial inflammatory insult is often not the reason for death from sepsis. It is, however, related to CARS-induced susceptibility to infection predisposing the patient to a secondary infection [14].

### 3.2. Infection and Immunosuppression in Burn Patients

Burn patients, especially those with burns greater than 20% of the body surface area, present with hyperinflammatory and hypermetabolic responses that can make management challenging. The SIRS criteria are fraught with many problems in diagnosis, as many of the clinical signs seen in burn patients present identically to the SIRS criteria, i.e., fever, tachycardia, and leukocytosis. Additionally, the CARS response, described above, poses a risk for complications in the post-resuscitative period. In order to prevent misdiagnosis, the American Burn Association developed a consensus to define sepsis more broadly. These consensus guidelines for sepsis in the burn patient are outlined elsewhere in this journal but include hyper- or hypothermia, progressive tachycardia, progressive tachypnea, thrombocytopenia, hyperglycemia, and inability to tolerate tube feeds [1]. Hogan et al. concluded that these criteria, however, did not correlate strongly with bacteremia. Their study described a sensitivity of 78.2% and specificity of 49.5% [15]. Notably, this study solely looked at cases of bacteremia based on the SIRS criteria and did not include sepsis from other causes.

The most common type of infection in the burn patient is wound infection. Colonization of burn wounds occurs shortly after the initial injury and should be considered as a source of infection in all burn patients. Gram-positive cocci, including methicillin-resistant *Staphylococcus aureus*, coagulase-negative *Staphylococci,* and *Enterococcus* species, are the usual culprits of wound colonization. Of Gram-negative organisms, *Pseudomonas* species are the most common colonizers, followed by *Klebsiella* species and *E. coli*, though these become more common at later stages of wound healing [16]. Burn wound colonization should not be treated with systemic antimicrobials, but rather with topical antimicrobials. Once the burn wound is infected, systemic antimicrobial therapy is warranted and should include coverage for the most common colonizing bacterial species. As previously mentioned, combat casualties with burn injury are at increased risk of developing infections with MDROs. Thus, even though resources may be limited in the austere environment, available antimicrobials (both systemic and topical) should include broad-spectrum agents that cover MDROs or fungal organisms.

In burn patients, the gut serves as another source of possible infection to remember. Shortly following a burn, splanchnic vasoconstriction and shunting leads to ischemia of the bowel. This, in turn, leads to mucosal atrophy. Animal models show that there is an increase in mucosal permeability that increases translocation of bacteria or bacterial products, creating a possible infection source [17,18]. Early enteral feedings remain effective at preserving mucosal integrity, but more studies are needed to determine other treatment options.

Management of burn wound infections is largely dependent on early prevention with wound cleaning. Colonization of these wound infections should not be treated with systemic antimicrobials; however, topical antibiotics can be used. Early recognition of infection remains difficult and pharmacologic management becomes complicated in the burn patient. Tailoring management relies on understanding the PK/PD changes seen in burn patients. Critically ill burn patients can demonstrate a change in renal clearance from hypermetabolism, but not always. In general, the literature suggests that there is a consensus about burn patients requiring higher dosing regimens. Huttner et al. concluded after a prospective observational study that there is an association between augmented renal clearance and reduced β-lactam trough levels. Of the patients in that study, trauma patients were more likely to demonstrate this augmented renal clearance and reduced antibiotic troughs [19]. Burn patients exhibit similar physiologic derangements as other trauma patients, but increasing dosing regimens may not be necessary [20,21,22].

Based on available research, Table 1 shows the recommended dosing of select antimicrobials in burn patients. The recommended dosing aims to optimize the characteristics of each agent while taking PK/PD alterations after burn injury into consideration. In general, for antibiotics with time-dependent killing (i.e., β-lactams), the recommended dose after burn injury is typically the high-end of ‘usual’ recommended dosing but infused over an extended period of time. Antibiotics with concentration-dependent killing (i.e., aminoglycosides) may need to be dosed more frequently due to increased/faster elimination after burn injury. Antibiotics that require the area-under-the-curve to minimum inhibitory concentration (AUC:MIC) ratio to be optimized (i.e., fluoroquinolones and vancomycin) may either require a higher dose or increased frequency after burn injury. It is important to note that the below recommendations do not apply to patients with impaired renal function or those who require renal replacement therapy. Wherever possible, therapeutic drug monitoring should be performed and antibiotic doses adjusted accordingly to achieve therapeutic levels. It is important to recognize that under-dosing antibiotics may lead to inadequate treatment and the development of drug-resistant organisms, and over-dosing antibiotics may lead to toxicity and end-organ damage.

## 4. Coagulation

### 4.1. Mechanisms of Coagulation

As a brief review, coagulation occurs in two distinct steps. First, initial hemostasis is driven by platelet adhesion and aggregation. Once the platelet plug has formed, secondary hemostasis can begin with the extrinsic and intrinsic coagulation pathways. The extrinsic pathway starts when tissue factor is exposed to blood, whereas the intrinsic pathway occurs when Factor XII is exposed to phospholipids on platelet membranes. Upon exposure to blood, tissue factor then complexes with Factor VIIa and converts Factor X to Factor Xa. In the intrinsic pathway, Factor XIIa activates Factor XI, then converting Factor IX to Factor IXa. This Factor IXa then converts Factor X to Factor Xa as well, converging to create the common coagulation pathway [4,24]. Enoxaparin blocks Factor Xa, preventing the formation of complexes with Factor Va, blocking the conversion of prothrombin to thrombin and thus the conversion of fibrinogen to fibrin. Heparin inactivates Factors IXa, Xa, XIa, XIIa, and plasmin, preventing the conversion of fibrinogen to fibrin.

### 4.2. Coagulopathy in Burn Patients

As mentioned above, burn patients are especially predisposed to developing coagulopathy. In the early post-burn phase (<48 h), this is likely due to an interplay from the hyperinflammatory response, fluid resuscitation, and trauma-induced coagulopathy [4,25]. As mentioned above, inflammation leads to activation of tissue factor and decreases natural anticoagulative pathways [24]. Large-volume resuscitation is often started after burn injury and can lead to hemodilution. Trauma leads to hypoperfusion in the tissues. Furthermore, burn trauma can create hypothermia, decreasing the efficacy of these coagulation proteins as body temperature begins to drop [25]. One retrospective study of 117 patients with ≥30% TBSA by Sherren et al. showed a statistically significant increase in TBSA associated with the presence of coagulopathy. About 40% of these patients presented with coagulopathy, suggesting that providers should be cognizant of this derangement early in the patient’s admission. Of note, fluid resuscitation was not significantly correlated with coagulopathy in these patients [26].

#### 4.2.1. Pathophysiology of Coagulopathy after Burn Injury

The pathophysiology of burn-induced coagulopathy is dependent on the phase of care. Early post-burn coagulopathy is marked by systemic activation and fibrinolysis disruption, while patients who survive to late-stage recovery tend to develop sepsis-induced coagulopathy [4]. Systemic activation is due to endothelial injury causing activation of tissue factor and release of pro-inflammatory cytokines. As expected, larger burns will damage more tissue, inducing more exposure of tissue factor and cytokines to the vasculature for activation of the coagulation cascade [26]. Fibrinolysis is seen in many trauma and sepsis patients. Ball et al. reviewed multiple studies investigating this disruption in fibrinolysis and found that some studies showed a decrease in anti-fibrinolytic proteins, such as antiplasmin, further showing a shift to a pro-coagulable state. However, there are some studies that suggest that patients demonstrate a hyperfibrinolytic state as well [4]. Following the initial post-burn resuscitative phase, patients are then susceptible to infection-based coagulopathy, likely due to immunosuppression from the CARS phenomenon. Sepsis and hyperinflammation can both increase binding to endothelial tissue factor through IL-1 and TNF-alpha, further promoting a pro-coagulable state. Additionally, several other pro-inflammatory cytokines, particularly IL-6, decrease fibrinolysis through indirect blockade of plasminogen conversion to plasmin. In burn patients, this cascade becomes deranged as coagulation factors can be depleted or dysfunctional, leading to a pro-coagulable state. The breakdown of clots also appears to be dysfunctional in hyperinflammatory states. The protein C pathway is downregulated by TNF-alpha and IL-1, further promoting thrombin formation [24]. Overall, it seems that there is a myriad of causes for coagulopathy in burn patients that interplay with each other to induce or exacerbate existing complications. Given this heterogenous data and lack of consensus, a patient’s individual markers may be a better indicator for management decisions. 

The importance of understanding coagulopathy in critically ill patients cannot be understated. Burn patients typically present with hypothermia due to heat loss from their wounds, acidosis often due to elevated lactate levels from decreased tissue perfusion, and coagulopathy due to the various aforementioned mechanisms. Classically, this is known as the lethal triad. Research shows that presence of one may imply the development of another arm in this triad. Sherren and colleagues performed a retrospective analysis and showed a significant correlation between patients presenting with coagulopathy and the presence of elevated serum lactate levels. Logically, further analysis of patients with coagulopathy showed increased mortality within 28 days, as earlier coagulopathy can be used as a predictor of mortality [26,27]. In a follow-up study, Sherren and colleagues looked at the incidence of this triad and its utility as a predictor of mortality. Unsurprisingly, there was a higher incidence of mortality; however, it was not associated with predicting mortality [28].

#### 4.2.2. Venous Thromboembolism after Burn Injury

One of the most worrisome complications in burn patients is venous thromboembolism (VTE). Burn patients are particularly susceptible due to their prolonged stasis from sedation, endothelial injury from their wounds, and hypercoaguability. These constitute all three criteria of Virchow’s triad. Additionally, given these alterations in physiology, it stands to reason that burn patients may need dose adjustments for adequate prophylaxis. Reduced cardiac output, hypoalbuminemia, and a low glomerular filtration rate in the early burn shock phase lower clearance and slow the rate of distribution into tissue. The hypermetabolic phase then results in increased clearance and subsequently lower serum drug levels [11,29]. Van Haren et al. concluded after a small, single-center, prospective observational trial that burn patients with ≥15% TBSA often did not present with hypercoagulability on admission but developed a hypercoagulable state 1 week after injury despite chemoprophylaxis of 5000 U heparin three times daily [30]. This suggests that VTE prophylaxis may be ineffective. Additionally, Sikora and Papp concluded that chemoprophylaxis does not prevent VTE as there was no difference in incidence of VTE when comparing patients on chemoprophylaxis vs. patients who were not [5]. In contrast, Liu et al. reported incidences of 0.84% for deep vein thrombosis and 0.19% for pulmonary embolisms using 5000 U subcutaneous heparin regimen every 8 h, in line with data from two large retrospective studies [31,32,33]. However, this study was performed at a single burn center with no monitoring labs, such as an anti-Factor Xa assay, to determine if patients were at accepted therapeutic levels.

Due to this heterogenous data, there is a lack of consensus on optimal chemoprophylaxis dosing. By monitoring therapies with an anti-Factor Xa assay, providers can tailor pharmacologic management to the patient’s specific needs. Lin et al. performed a prospective study and discovered that standard dosing for enoxaparin for VTE prophylaxis resulted in subtherapeutic levels (<0.2 U/mL) for acute burn patients, suggesting that this may be due to changes in absorption or increased renal elimination. Their analysis described a correlation between initial TBSA and necessary enoxaparin dose. However, the authors conceded that this was a small amount of data and that it is unknown if the dose–response curve will normalize once hypermetabolism ends or if BMI will significantly change dosing [29]. Other studies have investigated this enoxaparin dosing and concluded with similar results. Cronin et al. completed a retrospective review of the burn registry to monitor the efficacy of VTE chemoprophylaxis using peak anti-Xa levels and a dose adjustment strategy to maintain Xa levels at 0.2–0.4 IU/mL. At the conclusion of their study, there was a decreased incidence of VTE in the dose adjustment strategy group, although the authors reported that this study was insufficiently powered to show a statistically significant reduction. The important takeaway was that about 48% of patients required dose adjustments to meet therapeutic levels [34]. A survey of burn patients and VTE prophylaxis practices in the UK reported 5 out of 13 (38%) of the included institutions monitored Xa levels, all of which were burn centers. This represented an increase of 150% since 2019 [35]. Given the lack of consistent data due to alterations in clearance, monitoring anti-Xa levels and adjusting chemoprophylaxis accordingly may be prudent to ensure adequate VTE prophylaxis.

Other populations of burn patients may also require targeted dosing strategies based on monitoring anti-Xa levels, particularly pediatric and BMI-based dosing. Child dosing strategies of enoxaparin are also complicated by burn pathology and the data are often extrapolated from adult studies. Pediatric patients have a different physiology than adult patients at baseline and further derangements make preventing and managing complications difficult. One small study of 35 patients, performed by Brown et al., showed that 60% of pediatric burn patients had subtherapeutic anti-Factor Xa levels and that lower median age was associated with subtherapeutic levels [36]. This was a small study at a single institution; however, it demonstrates that more literature is needed on the subject. Similarly, enoxaparin is already known to need adjustments for weight-based dosing. Data from a study completed by Farrar et al. showed that trauma patients with higher total body weights were more often at subtherapeutic levels. The authors suggested, based on this data, that total body weight should be a factor in considering dosing regimens for VTE prophylaxis [37]. Burn patients represent a subset of trauma patients and may also need weight-based dosing strategies to optimize their care. Overall, burn patients have a complex overlay of mechanisms and physiology that increases their propensity to develop VTE. Additional patient factors such as sex, age, and weight all interplay with their course of treatment to optimize their care. Within burn care, monitoring assays can be helpful in preventing complications such as VTE during the hospital course.

## 5. Considerations for the Austere Environment

As discussed throughout this paper, burn injury results in significant physiologic and metabolic changes that affect drug dosing. As a result, pharmacokinetic monitoring and individualized dosing is recommended. However, given current technologies, this is likely not possible in the austere environment.

When preparing to care for a burn casualty for the first 7–10 days after injury, medications for analgesia, sedation, sepsis/infection (should be dictated by local antibiogram, if available), and general critical care support will be needed. Table 2 includes medications for consideration for use in the austere environment. Many of these medications are titrated to effect; specialized laboratory equipment for pharmacokinetic monitoring is not be needed. 

## 6. Future Research

Although there appears to be a positive trend of using monitoring assays, more evidence is needed in the burn population to determine best practices. Largely, burn literature remains sparse with specific and larger PK/PD studies, leading to reliance on provider discretion and inconsistencies across burn centers. More evidence is needed to create consensus recommendations for managing burn patients. Specifically, determining more sensitive and specific burn sepsis criteria will be useful for mitigating mortality risk. Point of care (POC) therapeutic drug monitoring devices represent a novel field of advancing burn care and randomized controlled trials are needed to determine their efficacy. Currently POC vancomycin level machines are in progress, but worldwide use has not been adopted. Finally, larger studies of monitoring assays in specific populations, such as electrical or caustic injury, within burn care are needed.

## 7. Conclusions and Summary

Burn patients represent a complex subset of trauma patients with physiologic derangements unique to their population. This alteration in physiology can affect the pharmacokinetics and pharmacodynamics of commonly used medications during their hospital stay. The literature shows that these dosage changes become complicated to manage and can lead to devastating complications, further challenging providers to provide optimal care. Monitoring assays, when available, should be used for providers to titrate dosing according to PD/PK changes and patient status.

## Figures and Tables

**Table 1:** Recommended dosing of select antimicrobials [23].

Antimicrobial	Recommended Dosing
Piperacillin/tazobactam	4.5 g IV q6h infused over 4 h
Cefepime	2 g IV q8h infused over 4 h
Ceftazidime	2 g IV q8h infused over 3 h
Imipenem/cilastatin	500 mg IV q6h *
Meropenem	1–2 g IV q8h *
Aztreonam	2 g IV q8h infused over 3 h OR 2 g IV q6h
Gentamicin	7 mg/kg (actual body weight) IV q24h ^+^
Tobramycin	10 mg/kg (actual body weight) IV q24h ^+^
Ciprofloxacin	400 mg IV q8h
Levofloxacin	750 mg IV q24h
Vancomycin	40–70 mg/kg/day in divided doses to achieve AUC:MIC 400–600 ^+^
Linezolid	600 mg IV q8h
Daptomycin	12 mg/kg IV q24h

* infuse over 3 h for infecting organisms with MIC > 2 mcg/mL. ^+^ may need more frequent dosing; therapeutic drug monitoring highly recommended.

**Table 2:** Proposed medications for use in the austere environment.

Analgesics and Sedatives [38]	
Acetaminophen (IV, PO)	Ketamine (IV, IO, IM, IN)
Hydromorphone (IV, IO)	Midazolam (IV/IO)
Fentanyl (IV, IO, IN, transmucosal)	Morphine (IV, IO, IN)
**Systemic Antimicrobials** [39]	
Ceftriaxone (IV/IO)	Metronidazole (IV/IO/PO)
Ertapenem (IV/IO)	Moxifloxacin (PO)
Levofloxacin (PO)	Vancomycin (IV/IO)
**General Critical Care Support**	
Balanced crystalloid solution (Lactated Ringers’, Plasmalyte)	VTE prophylaxis (enoxaparin, heparin)
Stress ulcer prophylaxis (pantoprazole IV, famotidine IV)	Vasopressors (vasopressin, norepinephrine)

## Data Availability

Not applicable.
